# Increased Yangtze finless porpoise presence in urban Wuhan waters of the Yangtze River during fishing closures

**DOI:** 10.1002/ece3.11247

**Published:** 2024-04-04

**Authors:** Zhi‐Tao Wang, Peng‐Xiang Duan, Tomonari Akamatsu, Ke‐Xiong Wang, Ding Wang

**Affiliations:** ^1^ School of Marine Science Ningbo University Ningbo Zhejiang China; ^2^ Institute of Hydrobiology Chinese Academy of Sciences Wuhan China; ^3^ Ocean Policy Research Institute the Sasakawa Peace Foundation Minato‐ku, Tokyo Japan

**Keywords:** boat traffic, hydrological regime, light intensity, passive acoustic monitoring, Wuhan, Yangtze finless porpoises

## Abstract

Wuhan, a highly urbanized and rapidly growing region within China's Yangtze Economic Zone, has historically been identified as a gap area for the critically endangered Yangtze finless porpoise (*Neophocaena asiaeorientalis asiaeorientalis*) based on daytime visual surveys. However, there has been a noticeable increase in porpoise sightings since 2020. This study employed passive acoustic monitoring to investigate porpoise distribution in Wuhan between 2020 and 2022. Generalized linear models were used to explore the relationship between shipping, hydrological patterns, light intensity, and porpoise biosonar activity. Over 603 days of effective monitoring, the daily positive rate for porpoise biosonar detection reached 43%, with feeding‐related buzz signals accounting for 55% of all porpoise biosonar signals. However, the proportion of minutes during which porpoise presence was detected was 0.18%, suggesting that while porpoises may frequent the area, their visits were brief and mainly focused on feeding. A significant temporal trend emerged, showing higher porpoise biosonar detection during winter (especially in February) and 2022. Additionally, periods without boat traffic correlated with increased porpoise activity. Hydrological conditions and light levels exhibited significant negative correlations with porpoise activity. Specifically, porpoise sonar detections were notably higher during the night, twilight, and new moon phases. It is highly conceivable that both fishing bans and COVID‐19 pandemic‐related lockdowns contributed to the heightened presence of porpoises in Wuhan. The rapid development of municipal transportation and shipping in Wuhan and resulting underwater noise pollution have emerged as a significant threat to the local porpoise population. Accordingly, it is imperative for regulatory bodies to effectively address this environmental stressor and formulate targeted protection measures to ensure the conservation of the finless porpoise.

## Graphical abstract

Yangtze finless porpoise was frequently sighted in the historical distribution gap and urban Wuhan. Fishing bans and COVID‐19 pandemic‐related lockdowns contributed to the heightened presence of porpoises in Wuhan.

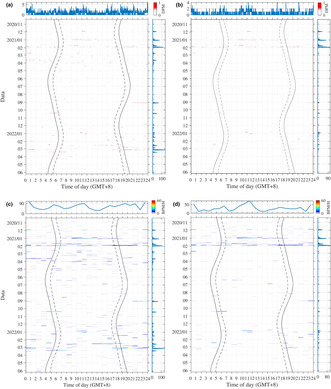

## 1 INTRODUCTION

Landscape alterations driven by urbanization pose significant harm to numerous species (Boakes et al., 2023). The resurgence of natural habitats within urbanized environments underscores a pivotal shift toward harmonizing human development with ecological conservation. This resurgence holds profound importance for the conservation of endangered species, as these habitats serve as critical sanctuaries and connectivity corridors essential for their survival and for the preservation of global biodiversity (Maxwell et al., 2020).

The Yangtze finless porpoise is the only freshwater porpoise in the world (Wang, 2009) and has been designated as “Critically Endangered” on the International Union for Conservation of Nature (IUCN) Red List since 2013 (Wang et al., 2013). Following the functional extinction of the baiji in 2006 (Turvey et al., 2007), the finless porpoise is now the only remaining cetacean in the Yangtze River, confined to the mainstem below Gezhouba Dam and connected lakes (Wang, 2009). Its habitat highly overlaps with human activity, making the species vulnerable to anthropogenic threats.

**FIGURE 1:**
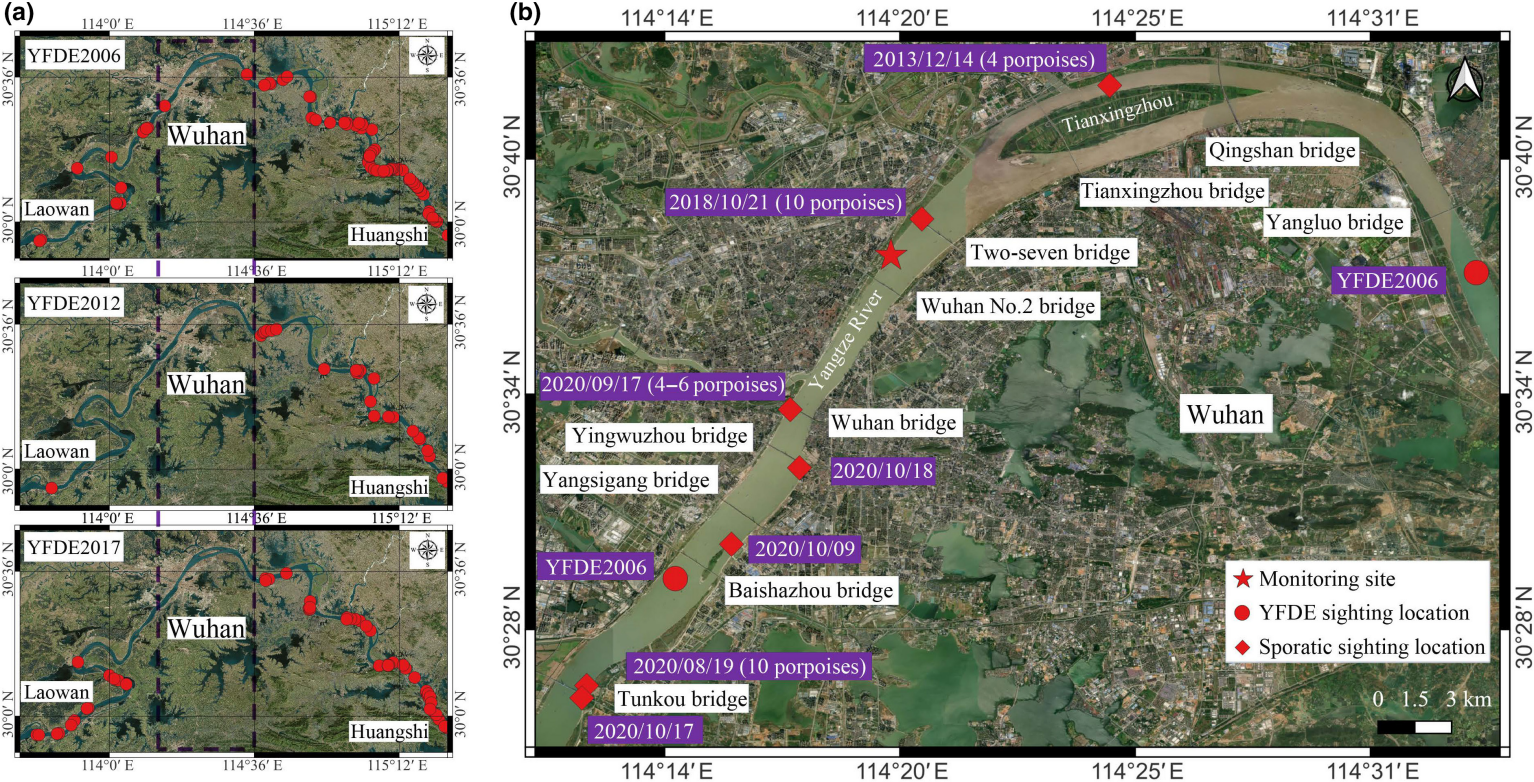
Sighting locations (red dot) of the Yangtze finless porpoise in the middle section of the mainstream of the Yangtze River between Laowan and Huangshi during the Yangtze freshwater dolphin expedition (YFDE) in 2006, 2012, and 2017, respectively, and a gap distribution zone of porpoise in Wuhan city was identified and highlighted with purple dashed box. B shows the gap distribution zone as identified by YFDE. The location where static acoustic monitoring was conducted was highlighted in red star mark and historical sighting of porpoise within the gap zone was also given in red diamond markers. On the basis of Google Maps, satellite map was made with QGIS software.

Wuhan, situated in central China, stands as the largest and most rapidly urbanized mega city in the region. Beyond its historical and cultural significance, it is also the central hub for economics, industry, transportation, and information (Cheng & Zhou, 2015; Tan et al., 2014). Often referred to as the “thoroughfare to nine provinces,” Wuhan serves as China's primary inland rail and road transportation nexus, where the Jingguang Railway and the Yangtze River intersect, running north to south and west to east (Tan et al., 2014). It holds a substantial population of around 12 million people as of 2020. Within the Yangtze Economic Zone and central China, Wuhan emerges as one of the most densely populated and rapidly growing areas. Nonetheless, information regarding the distribution of finless porpoises within Wuhan section of the middle Yangtze River remains scant. From 2006 to 2017, an extensive line transects visual survey conducted during daytime within the finless porpoise range of the Yangtze River revealed no presence of these creatures in the Wuhan region, particularly between the Baishazhou and Yangluo bridges (Figure 1). Accordingly, this region was classified as a gap area regarding finless porpoise distribution (Huang et al., 2020; Mei et al., 2014; Zhao et al., 2008). Nonetheless, sporadic sightings sourced from internet and television reports have been recorded, notably four porpoises near the Tianxinzhou Bridge on December 14, 2013, and approximately 10 porpoises downstream of the Two‐Seven Bridge on October 21, 2018. Notably, sightings increased since 2020, including around 10 porpoises observed near the Tunkou Bridge on August 19, 2020, and four to six porpoises upstream of the Wuhan Bridge on September 17, 2020. Further observations occurred near the Tunkou Bridge, Baishazhou Bridge, and Yingwuzhou Bridge on October 7th, 9th, and 18th, 2020, respectively (Figure 1) (Wang Zhi‐Tao, Unpublished data). With the surge in sightings, there is a pressing requirement for rigorous and ongoing monitoring to map out the distribution of porpoises in Wuhan, including their potential presence in the designated gap area at night. This information is vital for enhancing their protection and conservation efforts.

Cetaceans rely on biosonar for navigation, prey detection, and communication (Au & Benoit‐Bird, 2003; Madsen et al., 2023). The finless porpoise emits echolocation signals in click trains at a regular interval of 6.4 s (Akamatsu et al., 2007). These signals possess distinct attributes, featuring a narrow bandwidth (with a half power bandwidth of about 22 kHz), a high frequency (peaking at around 129 kHz), and a short duration (approximately 48 μs for −10 dB duration) (Fang et al., 2015). This unique sonar usage frequency and species‐specific signal traits make acoustic monitoring an effective approach for studying them (Akamatsu et al., 2001, 2010; Akamatsu, Matsuda, et al., 2005a). Passive acoustic monitoring, a noninvasive technique, has been extensively employed to track finless porpoises in the Yangtze River, significantly enhancing our insights into their distribution and habitat preferences (Akamatsu, Wang, et al., 2005b; Wang, Akamatsu, et al., 2015a, 2020a; Wang et al., 2014).

Herein, we sought to analyze the biosonar activity of Yangtze finless porpoises within Wuhan's region, employing passive acoustic monitoring. Furthermore, potential influencing factors, including anthropogenic elements like boat traffic and environmental variables like water level, water flux, and light intensity, were assessed to decipher their potential impact on the temporal presence of these porpoises.

## 2 MATERIALS AND METHODS

Passive acoustic monitoring was carried out at the Wuhan Marine Affairs Wharf (30°37′8″ N, 114°19′16″ E) in Wuhan, Hubei Province, located approximately 1100 km upstream from the Yangtze River's estuary (Figure 1). The acoustic recording spanned from October 12, 2020, to June 8, 2022.

### 2.1 Acoustic data collection

For acoustic data recording, an autonomous acoustic data logger known as F‐POD (Full waveform capture POrpoise Detectors, Chelonia Limited, UK) was utilized. This logger's hydrophone, operates within a frequency band of 20–160 kHz, was adept at capturing the biosonar signals of echo‐locating cetaceans, except for sperm whales. The click signals were sampled at a rate of 1 MHz. Various acoustic parameters, such as click duration, cycle count, wavelengths, amplitudes around the loudest cycle of the click, amplitude inflection count, period range, and click time, were documented (https://www.chelonia.co.uk/fpod_home_page.htm). The design of the F‐POD is informed by the extensive experience gained from C‐PODs deployed worldwide and integrates advancements in electronics. The F‐POD is designed to store a greater amount of higher‐quality data with each click, enhancing its capability for improved train detection and species classification (Ivanchikova & Tregenza, 2023).

The F‐POD was deployed vertically below the water surface, with the hydrophone at a depth of approximately 2 m. Retrieval of each F‐POD occurred every 2–3 months for data download. To ensure the examination of porpoise biosonar activity's temporal pattern, days with data gaps exceeding 1 h were excluded.

### 2.2 Acoustic data analysis

F‐POD data were processed using the F‐POD.exe software (Chelonia Limited, Mousehole, Cornwall, UK). KERNO‐F classifiers were employed to extract coherent click trains. Clicks with comparable spacing within trains were identified and categorized based on signal quality and type. Signal quality was classified as high, moderate, low, or of questionable quality, while signal categories encompassed narrow‐band high‐frequency clicks (signals resembling clicks from harbor porpoises), broadband high‐frequency clicks (signals resembling clicks from dolphin), boat sonar, and unclassified categories.

It is well‐established that finless porpoises generate buzz signals during attempted prey capture, although often it is uncertain whether the capture was successful. Buzz signals were characterized by trains of echolocation clicks with inter‐click intervals of less than 10 milliseconds (Akamatsu et al., 2010; Wang, Akamatsu, et al., 2015a; Wang et al., 2014). In this study, buzz signals served as proxies for cetacean attempted feeding behavior, facilitating the exploration of feeding patterns. Buzz signals were derived from narrow‐band high‐frequency clicks with inter‐click intervals of less than 10 milliseconds. Porpoise click type rates such as porpoise click detection positive rate per minute (DPRM), porpoise click train count per minute, buzz DPRM, and buzz count per minute were calculated.

### 2.3 Anthropogenic and environmental data

Anthropogenic and environmental factors were analyzed to assess the potential influence of boat traffic, hydrological conditions, light intensity, and lunar phases on porpoise sonar activity's temporal patterns. Anthropogenic activity was quantified using boat traffic. The F‐POD can log any type of boat that emits sonar signals, and boat traffic conditions were extracted from F‐POD records and represented as boat sonar detection positive rate per minute. Hydrological conditions encompassed water level and water flow. Hydrological data were sourced from the official water resources database (https://slt.hubei.gov.cn/sjfb/) using custom Python software. Light conditions consisted of diurnal and lunar cycles. Diel patterns analysis divided each day into three light periods: darkness, twilight, and daytime (Duan et al., 2023; Narganes Homfeldt et al., 2022). Lunar patterns were examined by extracting nighttime subset data and categorizing nighttime into three phases: new moon, quarter moon, and full moon (Wang, Nachtigall, et al., 2015b). Seasonal patterns divided dates into spring, summer, autumn, and winter. Local city‐specific diel phases, lunar phases, and seasonal divisions for Wuhan were obtained from https://www.timeanddate.com.

### 2.4 Statistical analysis

Data from days with less than 1 h of data loss were considered to investigate porpoise biosonar activity's temporal patterns. Generalized linear models (GLMs) were employed to explore associations between porpoise click type rates and factors such as boat traffic, water level, water flow, and light intensity. Due to collinearity between water level and water flow (paired sample correlation coefficient = .982, *p* < .001, Figure S1), only water level was integrated into the GLM model. A six‐way ANOVA full factorial design was utilized, with porpoise click type rates as dependent variables, boat traffic, diel, month, season, and year as fixed factors, and water level as a covariate. For lunar pattern analysis, nighttime subset data were analyzed using a six‐way ANOVA, with porpoise click type rates as dependent variables and boat traffic, lunar phase, month, season, and year as fixed factors, plus water level as a covariate. Variables and interaction terms with *p*‐values below .05 were retained in the final model. Post hoc multiple comparison tests were conducted using Tamhane's T2 method when significant differences were noted for fixed factors. Spearman's rank correlation was employed to determine correlations between porpoise biosonar detections, boat traffic, and hydrological conditions. Statistical analyses were conducted using SPSS version 26.0 (IBM Corp., Armonk, NY, USA) and a custom MATLAB R2018b script (The Mathworks, Natick, MA, USA).

## 3 RESULTS

**FIGURE 2:**
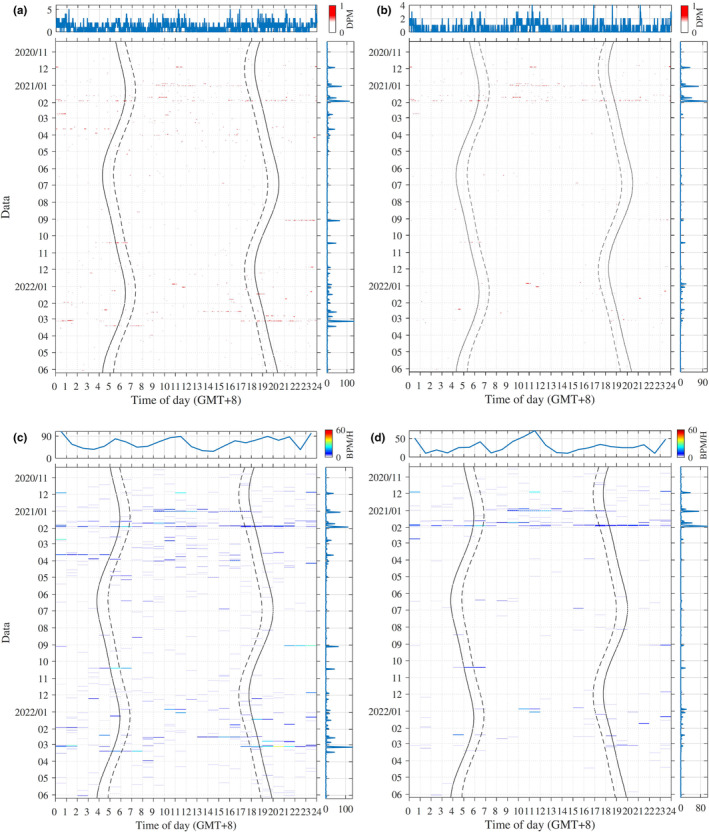
Porpoise click detection positive rate per minute (a), porpoise buzz detection positive rate per minute (b), porpoise click positive minute count per hour (c) and porpoise buzz‐positive minute count per hour (d) as a function of time of day (x‐axis) and date (y‐axis). Results are provided for every minute in (a) and (b) and for each hour in (c) and (d). The solid black lines indicate the start of dawn and the end of dusk. The dashed broken lines represent the end of dawn and the start of dusk. Line plots on the top denote the diel pattern over the investigation period. The line plot on the right side represents result in each day.

Nine equipment deployment rounds were conducted between 2020 and 2022, resulting in 603 effective monitoring days. Among these, porpoise sonar was detected on 257 days, yielding a proportion of porpoise‐positive days of 42.62%. Porpoise buzz signals were identified on 121 days, with a proportion of porpoise buzz‐positive days at 20.1%. Over the entire 868,165‐minute monitoring period, porpoise biosonar was observed for a combined total of 1521 min, representing a proportion of 0.18% porpoise‐positive minutes. Furthermore, 684 min contained porpoise buzz signals, constituting 0.08% of positive buzz minutes. During the 603 monitoring days, there were 5463 porpoise click trains and 3020 buzz signals, with porpoise buzz signals accounting for 55.3% of all porpoise click trains (Figure 2, Table 1).

**TABLE 1:** Results of porpoise biosonar detection.

Trial	Starting date	Starting time	Ending date	Ending time	Analyzed days	No. days with porpoise sonar	Daily porpoise sonar positive rate	No. porpoise click DPM	No. buzz DPM
1	10/12/2020	09:30	12/02/2020	09:25	50	18	36.00%	71	71
2	12/02/2020	09:40	02/04/2021	11:20	64	40	62.50%	414	402
3	02/04/2021	11:45	03/30/2020	11:35	54	28	51.85%	146	10
4	03/30/2020	12:00	06/07/2020	16:30	70	35	50.00%	113	8
5	06/07/2020	16:45	08/17/2021	12:00	71	18	25.35%	32	27
6	08/17/2021	12:15	10/15/2021	10:50	58	11	18.97%	123	30
7	10/15/2021	11:05	12/20/2021	16:15	67	32	47.76%	75	9
8	12/20/2021	16:40	03/19/2022	05:40	88	45	51.14%	497	120
9	03/19/2022	06:00	06/08/2021	15:40	81	30	37.04%	50	7
Sum					603	257	42.62%	1521	684

*Note*: Positive rate was obtained by dividing the number of days with porpoises biosonar detection by the whole analyzed days. Days with more than 1‐h data loss were not included for further statistical analysis. For each round of equipment deployment, days split between trials were assigned to the trial that had the greater proportion of hours. Date was given in mm/dd/yyyy, and time was given in hh:mm.

Abbreviations: DPM, detection positive minute.

**FIGURE 3:**
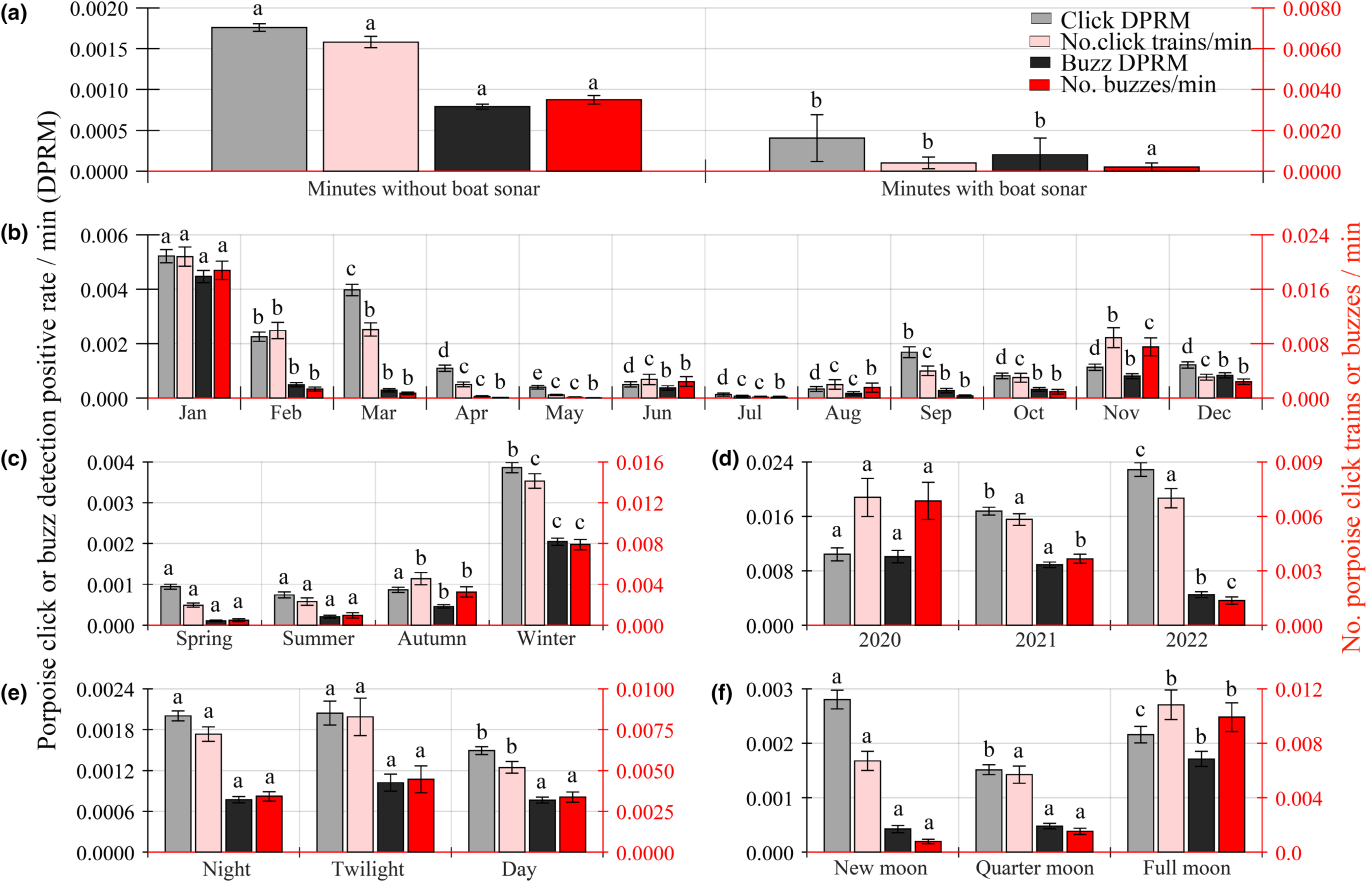
Variation in porpoise click type rates as a function of boat traffic (a), month (b), season (c), year (d), diel (e), and lunar (f) by a generalized linear model. Results are expressed as mean ± standard error of the mean. A subset of data was analyzed for lunar patterns at night. Error bars with identical letters represent post hoc multiple comparison tests that failed to detect a statistically significant difference (*p* > .05).

GLM analysis demonstrated significant monthly, seasonal, and annual temporal trends across all biosonar metrics (Table 2, Figure 3b–d). Monthly differences were observed for all porpoise click type rates, with significantly higher values noted in January. In February, March, and September, the porpoise click DPRM was significantly higher compared to other months, while no significant difference was observed between February and September. Porpoise click train count per minute in February, March, and November was significantly higher compared with other months, with no significant difference among these 3 months. Similarly, buzz DPRM in February, March, June, September, October, November, and December was significantly higher than in April, May, July, and August, while no significant difference existed among April, May, July, and August. November exhibited significantly more buzzes count per minute than other months, without a significant difference observed among the remaining months.

**TABLE 2:** Six‐way ANOVA of the effects of parameters (boat traffic * diel * month * season * year * water level) on the porpoise click detection positive rate per minute (DPRM), No. click trains/min, porpoise buzz DPRM and No. buzzes/min.

Source	Dependent variable	Type III sum of squares	Df	Mean square	*F*	Sig.
Corrected Model	Poroise click DPRM	2.39^a^	20	0.12	68.63	0.000
	No. click trains/min	36.26^b^	20	1.81	27.41	0.000
	Porpoise buzz DPRM	1.68^c^	20	0.08	107.56	0.000
	No. buzzes/min	35.03^d^	20	1.75	46.17	0.000
Intercept	Porpoise click DPRM	0.00	1	0.00	1.61	0.205
	No. click trains/min	0.38	1	0.38	5.70	0.017
	Porpoise buzz DPRM	0.01	1	0.01	13.11	0.000
	No. buzzes/min	1.69	1	1.69	44.64	0.000
Boat	Porpoise click DPRM	0.03	1	0.03	14.74	0.000
	No. click trains/min	0.34	1	0.34	5.10	0.024
	Porpoise buzz DPRM	0.00	1	0.00	4.00	0.046
	No. buzzes/min	0.07	1	0.07	1.88	0.171
Diel	Porpoise click DPRM	0.03	2	0.01	7.54	0.001
	No. click trains/min	0.55	2	0.27	4.15	0.016
	Porpoise buzz DPRM	0.01	2	0.00	6.17	0.002
	No. buzzes/min	0.18	2	0.09	2.33	0.097
Month	Porpoise click DPRM	0.69	11	0.06	36.10	0.000
	No. click trains/min	11.08	11	1.01	15.23	0.000
	Porpoise buzz DPRM	0.88	11	0.08	102.05	0.000
	No. buzzes/min	20.61	11	1.87	49.39	0.000
Season	Porpoise click DPRM	0.18	3	0.06	34.01	0.000
	No. click trains/min	2.03	3	0.68	10.24	0.000
	Porpoise buzz DPRM	0.07	3	0.02	30.67	0.000
	No. buzzes/min	0.80	3	0.27	7.03	0.000
Year	Porpoise click DPRM	0.03	2	0.01	7.59	0.001
	No. click trains/min	1.60	2	0.80	12.11	0.000
	Porpoise buzz DPRM	0.18	2	0.09	112.50	0.000
	No. buzzes/min	5.42	2	2.71	71.41	0.000
Water level	Porpoise click DPRM	0.01	1	0.01	3.59	0.058
	No. click trains/min	0.27	1	0.27	4.13	0.042
	Porpoise buzz DPRM	0.01	1	0.01	10.54	0.001
	No. buzzes/min	1.64	1	1.64	43.35	0.000
Error	Porpoise click DPRM	1515.94	868,299	0.00		
	No. click trains/min	57,452.36	868,299	0.07		
	Porpoise buzz DPRM	681.77	868,299	0.00		
	No. buzzes/min	32,940.46	868,299	0.04		
Total	Porpoise click DPRM	1521.00	868,320			
	No. click trains/min	57,523.00	868,320			
	Porpoise buzz DPRM	684.00	868,320			
	No. buzzes/min	32,986.00	868,320			
Corrected total	Porpoise click DPRM	1518.34	868,319			
	No. click trains/min	57,488.63	868,319			
	Porpoise buzz DPRM	683.46	868,319			
	No. buzzes/min	32,975.50	868,319			

^a^ *R* ^2^ = .002 (Adjusted *R* ^2^ = .002).

^b^ *R* ^2^ = .001 (Adjusted *R* ^2^ = .001).

^c^ *R* ^2^ = .002 (Adjusted *R* ^2^ = .002).

^d^ *R* ^2^ = .001 (Adjusted *R* ^2^ = .001).

All four biosonar characteristics demonstrated significant seasonal variations, with higher detections in winter compared with other seasons. Porpoise click train count per minute, buzzes DPRM, and buzz count per minute in autumn were significantly higher than in spring and summer, while spring and summer did not exhibit a significant difference in detections (post hoc Tukey's HSD; *p* > .05) (Table 2 and Figure 3c).

Annual differences were evident in all biosonar metrics, except for porpoise click train count per minute (post hoc Tukey's HSD; *p* > .05) (Table 2 and Figure 3d). In 2022, porpoise click DPRM was significantly higher than 2020 and 2021, whereas buzz DPRM and buzz count per minute exhibited the opposite trend (Table 2 and Figure 3d). A positive correlation was observed between porpoise click trains and porpoise buzz detections (Spearman's rho, *p* < .001, *n* = 868,320).

### 3.1 Boat traffic

**FIGURE 4:**
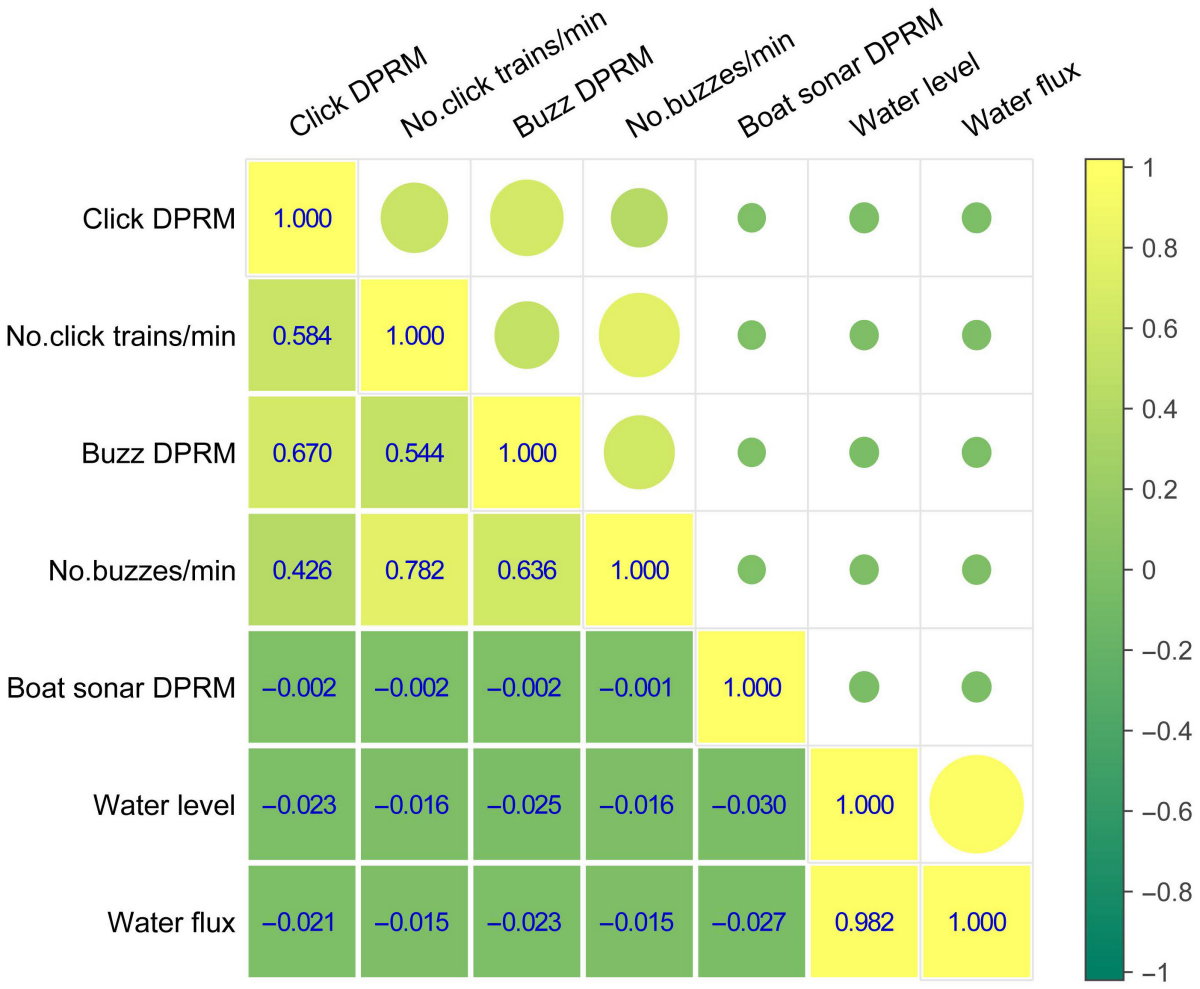
Spearman's rho correlation of porpoise biosonar detection, boat traffic and hydrological regime. Correlation coefficient was presented for each pairwise linear correlation. DPRM, positive detection rate per minute.

GLM results indicated that porpoise click DPRM, porpoise click train count per minute and buzzes DPRM during periods with boat traffic were significantly lower than those without boat traffic. However, buzz count per minute did not significantly differ between periods with and without boat traffic (Table 2 and Figure 3a). A significant negative correlation was observed between porpoise sonar detections and boat sonar detections (Spearman's rho, *p* < .05, *n* = 868,320) (Figure 4).

### 3.2 Water flow and water level

Water flow and water level exhibited a clear dynamic pattern (Figure S1). GLM results revealed substantial effects of water flow on all porpoise click type rates (Table 2). Since water flux and water level exhibited collinearity and high correlation (Figure 4), water level was anticipated to have a similar impact on all porpoise click type rates as water flow. Porpoise biosonar detections exhibited a significant negative correlation with both water level and water flow (Spearman's rho, *p* < .001, *n* = 868,320) (Figure 4).

### 3.3 Diel and lunar patterns

Significant diel pattern variations were noted for porpoise click DPRM and porpoise click train count per minute, while no significant diel patterns were observed for buzz DPRM and buzz count per minute (Table 2 and Figure 3e). Notably, nocturnal and twilight‐time biosonar activity exceeded daytime activity, with no significant difference observed in porpoise click parameters between nocturnal and twilight periods (post hoc Tukey's HSD; *p* > .05). Significant differences were observed in lunar patterns for all porpoise click type rates, except for porpoise click train count per minute between the new moon and quarter moon (post hoc Tukey's HSD; *p* > .05) and buzz rates between the new moon and quarter moon (post hoc Tukey's HSD; *p* > .05) (Table S1 and Figure 3f). Specifically, porpoise click DPRM during the new moon was significantly higher than during the quarter moon and full moon (post hoc Tukey's HSD; *p* < .05) (Table S1 and Figure 3f). Additionally, porpoise buzz activity was significantly higher during the full moon compared with the new moon and quarter moon (post hoc Tukey's HSD; *p* < .05) (Table S1 and Figure 3f).

## 4 DISCUSSION

In this study, the high daily porpoise biosonar detection rate, substantial proportion of feeding‐correlated buzz signals, and low proportion of positive minutes imply that porpoises may visit frequently, but their stays are brief and primarily centered around feeding activities within the monitoring site. Throughout the continuous 3‐year monitoring period of the present study, the proportion of porpoise‐positive days (43%) was lower than that reported between the confluence of Poyang Lake and the Yangtze River (93%) (Duan et al., 2023) and in the middle section of the Yangtze River downstream of the Gezhouba Dam (ranging from 54% to 73%) (Wang et al., 2024). Notably, feeding‐correlated buzz signals (constituted 55% of all porpoise biosonar) were higher than the confluence of Poyang Lake and the Yangtze River (23%) (Duan et al., 2023) but lower than the middle section downstream of the Gezhouba Dam (ranging from 62% to 81%) (Wang et al., 2024). Furthermore, the proportion of minutes categorized as porpoise‐positive (0.18%) was lagged behind the confluence of Poyang Lake and the Yangtze River (3.80%) (Duan et al., 2023) and the middle section downstream of the Gezhouba Dam (ranging from 0.36% to 14.64%) (Wang et al., 2024). Harbor porpoises (*Phocoena phocoena*) were also frequently subjected to acoustic monitoring in the New York–New Jersey harbor estuary, which stands as one of the world's most urbanized estuaries (Rekdahl et al., 2023). Additionally, an extraordinary influx of humpback whales (*Megaptera novaeangliae*) into the highly urbanized San Francisco Bay commenced in 2016 (Markowitz et al., 2024).

### 4.1 Water level and flow

Our investigation found that both water level and water flux had a significant impact on the biosonar activity of finless porpoises, consistent with previous findings reported on the confluence between Poyang Lake and the Yangtze River (Duan et al., 2023) and in the middle section of the Yangtze River downstream of the Gezhouba Dam (Wang et al., 2024). One potential explanation for the heightened detection of porpoise biosonar during the dry season (particularly between November and May, see Figure S1) may be attributed to the reduced water area in the Yangtze River and the contraction of the porpoise's distribution range during this period. However, the mechanism underlying this phenomenon requires more in‐depth investigation.

### 4.2 Diel, lunar, and temporal patterns

Our investigation revealed distinct patterns in cetacean biosonar activity, notably a consistent trend of greater acoustic activity during nighttime, aligning with findings from previous studies (Caruso et al., 2017; Wang, Nachtigall, et al., 2015b). This finding corresponds with observations in the Pengze area of the Yangtze River (Wang et al., 2014) as well as in the confluence region of Poyang Lake and the Yangtze River (Duan et al., 2023) and the middle section downstream of the Gezhouba Dam (Wang et al., 2024). In contrast, our study reported significantly higher porpoise biosonar activity during new moon periods than quarter moon and full moon phases. This contradicts findings from the confluence area of Poyang Lake and the Yangtze River (Duan et al., 2023) and the middle section downstream of the Gezhouba Dam (Wang et al., 2024). Additionally, we observed elevated biosonar activity during winter compared with other seasons, which mirrors the results from the confluence area of Poyang Lake and the Yangtze River (Duan et al., 2023) but contrasts with the findings downstream of the Gezhouba Dam (Wang et al., 2024). Interestingly, our study indicated a gradual increase in porpoise biosonar activity from 2020 to 2022. This trend contrasts with reports on the confluence region of Poyang Lake and the Yangtze River (Duan et al., 2023) and the middle section downstream of the Gezhouba Dam (Wang et al., 2024). A plausible explanation for these divergent temporal patterns in biosonar activities could involve porpoise migration between different regions of the Yangtze River. However, to gain a comprehensive understanding of this phenomenon, further investigation is warranted.

### 4.3 Boat traffic

A significant negative correlation between porpoise biosonar detection and boat traffic was observed, consistent with findings on the confluence of Poyang Lake and the Yangtze River (Duan et al., 2023) and the middle section downstream of the Gezhouba Dam (Wang et al., 2024). The Yangtze River holds the world's top inland shipping capacity since 2005, with significant port growth. Wuhan, as the shipping hub of the middle Yangtze River, possesses one of the top three largest container ports on the Yangtze River (Zhou et al., 2023). The bustling shipping activities inevitably introduce substantial underwater noise pollution into the Yangtze River (Wang, Akamatsu, et al., 2020a; Wang, Duan, Akamatsu, et al., 2021a). It is now understood that freshwater cetaceans are susceptible to noise pollution's effects (Wang, Duan, Wang, & Wang, 2021b). Despite much of the underwater noise in the Yangtze River comprising low frequencies (Wang, Akamatsu, et al., 2020a; Wang, Duan, Akamatsu, et al., 2021a) which fall beyond the recognized hearing range of finless porpoises (Wang, Li, et al., 2020b), exposure to inaudible low‐frequency sounds can still alter inner ear function, rendering them temporarily more prone to damage (Kugler et al., 2014; Williams, 2014). Given the foreseeable lack of improvement in navigation conditions on the Yangtze River, it is imperative to regulate and control boat traffic for the welfare of the cetaceans.

### 4.4 Factors influencing the porpoise distribution in Wuhan

Metabolic demands have been studied in other porpoise species, such as the harbor porpoise, where continuous feeding behavior has been documented (Wisniewska et al., 2016). Prey distribution is widely believed to influence the presence of cetaceans (Gilles et al., 2016; Rekdahl et al., 2023; Wang, Nachtigall, et al., 2015b; Wisniewska et al., 2016). In the Yangtze River, prey availability also shapes the distribution of finless porpoises (Wang, Akamatsu, et al., 2015a; Wang et al., 2014). The severe overfishing in China's Yangtze River has led to a decline in its biological integrity index to a “no fish” level (Ministry of Agriculture and Rural Affairs, 2021). The discovery of finless porpoises in regions with ample food resources highlights the association between prey availability and their distribution (Kimura et al., 2012). The implementation of a strictly enforced 10‐Year Fishing Ban on all aquatic nature reserves and germplasm resource reserves in the mainstream and crucial tributaries of the Yangtze River since January 1, 2020, with an extension to cover the entire area starting January 1, 2021 (Ministry of Agriculture and Rural Affairs, 2019) has aimed to address this issue. In Wuhan, the fishing ban's enforcement (from July 1, 2020, approximately 6 months earlier than in other non‐reserve areas of the Yangtze River), might have contributed to the alleviation of the decline in fisheries resource. This could have encouraged porpoises from adjacent waters to migrate to the Wuhan region.

Sustainable management of both porpoises and local fishermen necessitates a comprehensive year‐by‐year approach. The 10‐Year Fishing Ban has demonstrated benefits for porpoise populations in the Yangtze River. This involves assessing fish stocks species‐by‐species, permitting fishing once species reach a healthy state, and adopting practices like leaving smaller fish to sustain populations, regulated through mesh size restrictions.

To minimize the spread of COVID‐19, stringent pandemic lockdown measures were enforced in Wuhan from January 23 to April 8, 2020. This entailed the temporary cessation of all commercial activities, suspension of non‐essential travel including boat traffic and transport through river‐crossing tunnels, bridges, and other modes of transportation, along with mandatory home quarantine for individuals. These measures collectively led to a notable decrease in anthropogenic noise pollution, particularly stemming from boat traffic (Lecocq et al., 2020) and could further encouraged porpoises from adjacent waters to migrate to the Wuhan region. Moreover, strategies such as limiting nighttime ship traffic volume and introducing electric propulsion ships could facilitate coexistence between porpoises and the urban environment (Wang, Duan, Akamatsu, et al., 2021a).

Additionally, the elusive nature of finless porpoises, with their swift surfacing for breaths and lack of dorsal fins, has made visual observation in the busy navigation context of the Wuhan region challenging. Previous daytime visual surveys that identified Wuhan as a gap in the distribution of porpoises within the Yangtze River cannot be directly compared to continuous passive acoustic monitoring throughout the day, as our study reveals that the animals are more frequently detected during nighttime. Prior to our study, the lack of long‐term visual or acoustic porpoise monitoring in Wuhan made it challenging to rule out the possibility of porpoises occasionally visiting the region, particularly at nighttime, albeit less frequently.

### 4.5 Conservation and management implications

Within the dynamic Yangtze Economic Zone, Wuhan is a rapidly growing and densely urbanized area with highly developed municipal transportation systems. This urban landscape features 11 cross‐river bridges (Figure 1) and 5 river‐crossing tunnels within a span of approximately 59 km between the Junshan and Yangluo regions. Plans for an additional two river‐crossing tunnels are underway. The movement of vehicles and trains across these bridges and through tunnels can trigger vibrations and noise that reverberate through the water (Song et al., 2020, 2023), potentially affecting aquatic species such as porpoises (Kight & Swaddle, 2011). Furthermore, the transportation‐related noise adds to the already significant underwater noise pollution from bustling vessel traffic (Wang, Akamatsu, et al., 2020a; Wang, Duan, Akamatsu, et al., 2021a). The escalation of anthropogenic noise demands vigilant consideration as a prominent form of environmental pollution. Accountability for noise reduction falls on governments, and legislative bodies must institute more effective regulations to mitigate this environmental stressor. Implementing no‐navigation zones within the core habitats of finless porpoises becomes imperative. Vessel noise, which can disrupt foraging in wild harbor porpoises (Wisniewska et al., 2018), can be alleviated through minor reductions in cargo vessel speed (Findlay et al., 2023). Thus, exploration and implementation of ecologically friendly vessel navigation speeds and traffic flux should become urgent priorities for the Yangtze River.

The increasing incidence of porpoise biosonar detection in Wuhan during the fishing ban in this study could be attributed to various factors, including the growing population size (Wang Ding, Unpublished data), availability of prey, factors related to the fishing ban (such as decreased use of fishing gear and bycatch, increased prey availability, and reduced fishing boat traffic), influences stemming from COVID‐19 lockdowns (such as decreased boat traffic and reduced underwater noise pollution), or a combination of these factors. It is crucial to acknowledge that this study assessed the species distribution in Wuhan utilizing only a single hydrophone at one site. A more extensive array is essential for a comprehensive and fine‐scale evaluation of animal presence in the area.

## 5 CONCLUSION

Herein, we employed passive acoustic monitoring to explore local porpoise biosonar activities within Wuhan, a region lacking comprehensive information about finless porpoises. Our three‐year monitoring endeavor yielded baseline insights into the contemporary presence of porpoises in Wuhan. The high daily positive detection rate of porpoise biosonar (43%) and the low proportion of porpoise‐positive minute (0.18%) suggests that porpoises frequent the area, but their presence tends to be brief. A pronounced temporal trend marked by monthly, seasonal, and annual fluctuations was evident in porpoise biosonar detections. Implementing a 10‐year fishing ban in the Wuhan section of the Yangtze River since 2020 and the pandemic lockdown may have fostered the presence of porpoises in the region. Emphasis should be placed on the fact that this study marks the first long‐term acoustic deployment in the study area. As such, it is possible that habitat use patterns of porpoises may have been overlooked when employing traditional cetacean surveys.

## AUTHOR CONTRIBUTIONS

**Zhi‐Tao Wang:** Conceptualization (lead); data curation (lead); formal analysis (lead); funding acquisition (lead); investigation (lead); methodology (lead); project administration (lead); resources (lead); software (lead); supervision (lead); validation (lead); visualization (lead); writing – original draft (lead); writing – review and editing (lead). **Peng‐Xiang Duan:** Data curation (equal); formal analysis (equal); validation (equal); visualization (equal); writing – original draft (equal); writing – review and editing (equal). **Tomonari Akamatsu:** Data curation (equal); formal analysis (equal); methodology (equal); validation (equal); writing – review and editing (equal). **Ke‐Xiong Wang:** Conceptualization (equal); data curation (equal); funding acquisition (equal); writing – review and editing (equal). **Ding Wang:** Conceptualization (equal); funding acquisition (equal); project administration (equal); writing – original draft (equal); writing – review and editing (equal).

## FUNDING INFORMATION

This work was supported by the Ningbo University's Talent Introduction Research Startup Funding (Grant No. ZX2023000154) and the Science and Technology Service Network Initiative of the Chinese Academy of Sciences (KFJ‐STS‐QYZD‐2021‐27‐001).

## CONFLICT OF INTEREST STATEMENT

The authors declare that they have no known competing financial interests or personal relationships that could have appeared to influence the work reported in this paper.

## Supporting information


Figure S1.

Table S1.

## Data Availability

The data that support the findings of this study are openly available in Dryad at: https://datadryad.org/stash/share/yzALc6Zl1OAYdxeQ2omNJi_u8EdMcf9nhZWZ00ooFAo.
