# Supplementary material for: Increased Yangtze finless porpoise presence in urban Wuhan waters of the Yangtze River during fishing closures
Source: Ecol Evol. 2024 Apr 4;14(4):e11247. doi: 10.1002/ece3.11247 (PMC10994980; doi:10.1002/ece3.11247)
Supplement: Supplementary file 1 — Figure S1. [file ECE3-14-e11247-s001.doc]

Figure S1 Water level and water flux during the passive acoustic monitoring period in Wuhan
