# Supplementary material for: Increased Yangtze finless porpoise presence in urban Wuhan waters of the Yangtze River during fishing closures
Source: Ecol Evol. 2024 Apr 4;14(4):e11247. doi: 10.1002/ece3.11247 (PMC10994980; doi:10.1002/ece3.11247)
Supplement: Supplementary file 2 — Table S1. [file ECE3-14-e11247-s002.doc]

**Table s1.** Six-way ANOVA of the effects of parameters (boat traffic * lunar * month * season * year * water level) on the porpoise click detection positive rate per minute (DPRM), No. click trains / min, porpoise buzz DPRM and No. buzzes / min.

| Source |  | Type III Sum of Squares | df | Mean Square | F | Sig. |
| --- | --- | --- | --- | --- | --- | --- |
| Corrected Model | Click DPRM | 1.698a | 20 | 0.085 | 42.567 | 0.000 |
|  | No. click trains/min | 20.447b | 20 | 1.022 | 13.573 | 0.000 |
|  | Buzz DPRM | 0.608c | 20 | 0.030 | 39.490 | 0.000 |
|  | No. buzzes/min | 18.330d | 20 | 0.917 | 29.925 | 0.000 |
| Intercept | Click DPRM | 0.016 | 1 | 0.016 | 7.862 | 0.005 |
|  | No. click trains/min | 0.004 | 1 | 0.004 | 0.054 | 0.816 |
|  | Buzz DPRM | 0.004 | 1 | 0.004 | 4.636 | 0.031 |
|  | No. buzzes/min | 0.579 | 1 | 0.579 | 18.912 | 0.000 |
| Boat | Click DPRM | 0.020 | 1 | 0.020 | 10.035 | 0.002 |
|  | No. click trains/min | 0.215 | 1 | 0.215 | 2.848 | 0.091 |
|  | Buzz DPRM | 0.002 | 1 | 0.002 | 2.341 | 0.126 |
|  | No. buzzes/min | 0.035 | 1 | 0.035 | 1.155 | 0.282 |
| Lunar | Click DPRM | 0.087 | 2 | 0.044 | 21.890 | 0.000 |
|  | No. click trains/min | 1.308 | 2 | 0.654 | 8.685 | 0.000 |
|  | Buzz DPRM | 0.095 | 2 | 0.047 | 61.614 | 0.000 |
|  | No. buzzes/min | 4.634 | 2 | 2.317 | 75.655 | 0.000 |
| Season | Click DPRM | 0.125 | 3 | 0.042 | 20.817 | 0.000 |
|  | No. click trains/min | 0.991 | 3 | 0.330 | 4.388 | 0.004 |
|  | Buzz DPRM | 0.003 | 3 | 0.001 | 1.353 | 0.255 |
|  | No. buzzes/min | 0.132 | 3 | 0.044 | 1.441 | 0.229 |
| Month | Click DPRM | 0.814 | 11 | 0.074 | 37.098 | 0.000 |
|  | No. click trains/min | 6.790 | 11 | 0.617 | 8.195 | 0.000 |
|  | Buzz DPRM | 0.292 | 11 | 0.027 | 34.552 | 0.000 |
|  | No. buzzes/min | 8.475 | 11 | 0.770 | 25.157 | 0.000 |
| Year | Click DPRM | 0.010 | 2 | 0.005 | 2.511 | 0.081 |
|  | No. click trains/min | 0.281 | 2 | 0.141 | 1.866 | 0.155 |
|  | Buzz DPRM | 0.053 | 2 | 0.026 | 34.244 | 0.000 |
|  | No. buzzes/min | 2.224 | 2 | 1.112 | 36.308 | 0.000 |
| Water level | Click DPRM | 0.020 | 1 | 0.020 | 10.112 | 0.001 |
|  | No. click trains/min | 0.000 | 1 | 0.000 | 0.002 | 0.964 |
|  | Buzz DPRM | 0.003 | 1 | 0.003 | 3.955 | 0.047 |
|  | No. buzzes/min | 0.574 | 1 | 0.574 | 18.726 | 0.000 |
| Error | Click DPRM | 736.820 | 369393 | 0.002 |  |  |
|  | No. click trains/min | 27822.256 | 369393 | 0.075 |  |  |
|  | Buzz DPRM | 284.173 | 369393 | 0.001 |  |  |
|  | No. buzzes/min | 11313.345 | 369393 | 0.031 |  |  |
| Total | Click DPRM | 740.000 | 369414 |  |  |  |
|  | No. click trains/min | 27862.000 | 369414 |  |  |  |
|  | Buzz DPRM | 285.000 | 369414 |  |  |  |
|  | No. buzzes/min | 11336.000 | 369414 |  |  |  |
| Corrected Total | Click DPRM | 738.518 | 369413 |  |  |  |
|  | No. click trains/min | 27842.702 | 369413 |  |  |  |
|  | Buzz DPRM | 284.780 | 369413 |  |  |  |
|  | No. buzzes/min | 11331.675 | 369413 |  |  |  |

1. R Squared = .002 (Adjusted R Squared = .002)
2. R Squared = .001 (Adjusted R Squared = .001)
3. R Squared = .002 (Adjusted R Squared = .002)
4. R Squared = .002 (Adjusted R Squared = .002)
